# Dataset on genetic variation and trait association in cheeseweed (*Malva parviflora* L*.*) genotypes for agronomic traits

**DOI:** 10.1016/j.dib.2022.108651

**Published:** 2022-10-11

**Authors:** Md. Marufur Rahman, Md. Ashraful Alam, Hasibul Hasan, Mirana Akhter Sumi, Md. Maksudul Haque

**Affiliations:** a Regional Station Rangpur, Bangladesh Institute of Research and Training on Applied Nutrition (BIRTAN), Rangpur, Bangladesh; b Plant Breeding Division, Spices Research Center, Bangladesh Agricultural Research Institute (BARI), Bogura, Shibganj, Bangladesh; c Department of Agriculture, Rabindra Maitree University (RMU), Kustia, Bangladesh; d Regional Agricultural Research Institute, Bangladesh Agricultural Research Institute (BARI), Moulvibazar, Akbarpur, Bangladesh; e Head Office, Bangladesh Institute of Research and Training on Applied Nutrition (BIRTAN), Narayanganj, Araihazar, Bangladesh

**Keywords:** Genetic variability, Heritability (H), Genetic advance (GA), Genotypic co-efficient of variation (GCV), Phenotypic co-efficient of variation (PCV)

## Abstract

In order to analyze genetic variability, heritability, genetic advance and trait associations in *Malva parviflora* genotypes for agronomic traits, this paper presented a dataset. Seven agronomic traits variation and genetic parameters, including phenotypic and genotypic variance, genotypic and phenotypic coefficients of variation, broad-sense heritability, genetic advance, traits association, principal component analysis, and heatmap analysis were performed based on phenotypic data. Excel, PBtools, STAR, and R applications were used to analyze the data. There was substantial variation for the traits as revealed by descriptive statistics and variance analysis. Graphical presentation showed for principal component analysis and heatmap analysis. Researchers can use this dataset as guide to their plan for improvement this crop as leafy vegetables.

**Specifications Table**

**Table:** 

Subject	Agricultural Sciences
Specific subject area	Agronomy, Plant breeding
Type of data	Table and Figure
How the data were acquired	Variability and character association analysis
Data format	Analyzed
Description of data collection	Measurements from marketable mature plants were used to collect data on seven agronomic parameters. For data collection, a total of 3 plants were chosen from each replication. The number of leaves were manually gathered and counted. The average of the plant height (cm), petiole length (cm), leaf length (cm), leaf breadth (cm), root length (cm), and plant weight (g) were computed.
Data source location	Institution: Bangladesh Institute of research and Training on Applied Nutrition (BIRTAN), Regional Station, Rangpur, Bangladesh City/Town/Region: Rangpur Country: Bangladesh Latitude and longitude (and GPS coordinates, if possible) for collected samples/data: 25° 44′ 47.90″ N and 89° 15′ 5.98″ E
Data accessibility	Repository name: Mendeley Data Data identification number: 10.17632/mcxv7d6xgk.4 Direct URL to data: https://data.mendeley.com/datasets/mcxv7d6xgk/4

## Value of the Data

• The data provides insight genetic advance, heritability, variability and relationships of agronomic traits.
• Researchers working in agronomy, leafy vegetables, and plant breeding can benefit from these data.
• The detail in the data is important for researchers choosing the best heritable traits for design crop development programs.
• Cheeseweed (*Malva parviflora* L*.*) is a very well-known orphan leafy vegetable with considerable prospects as a crop in terms of nutritional content. It might offer a good source of nutrients to farmers in Rangpur region, Bangladesh.

## 1 Data Description

The raw data of the experiment is available at mendeley data https://data.mendeley.com/datasets/mcxv7d6xgk/4 [1]

### 1.1 The Analysis of Variance and Frequency Distribution of Traits

**Fig. 1:**
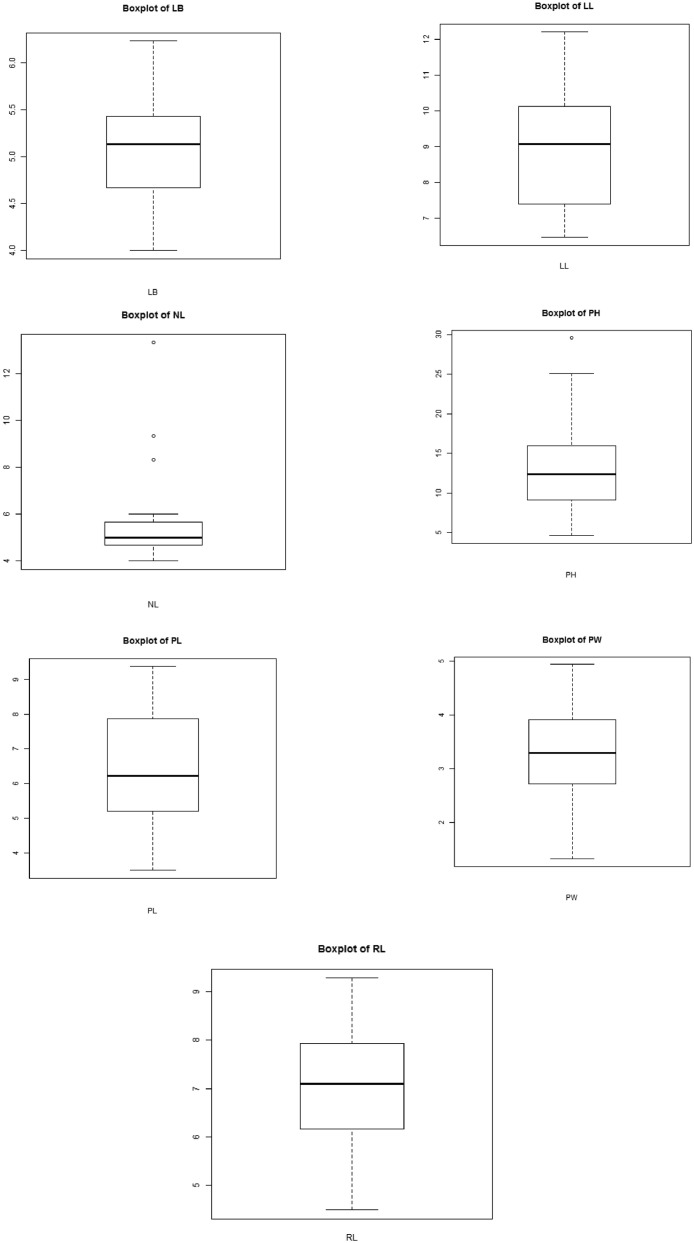
Box plots of the measured traits. Keys to abbreviations: PH= Plant height (cm), NL= Number of leaves, PL= Petiole length (cm), LL= Leaf length (cm), LB=Leaf breadth (cm), RL=Root length (cm), and PW= Plant weight (g).

**Fig. 2:**
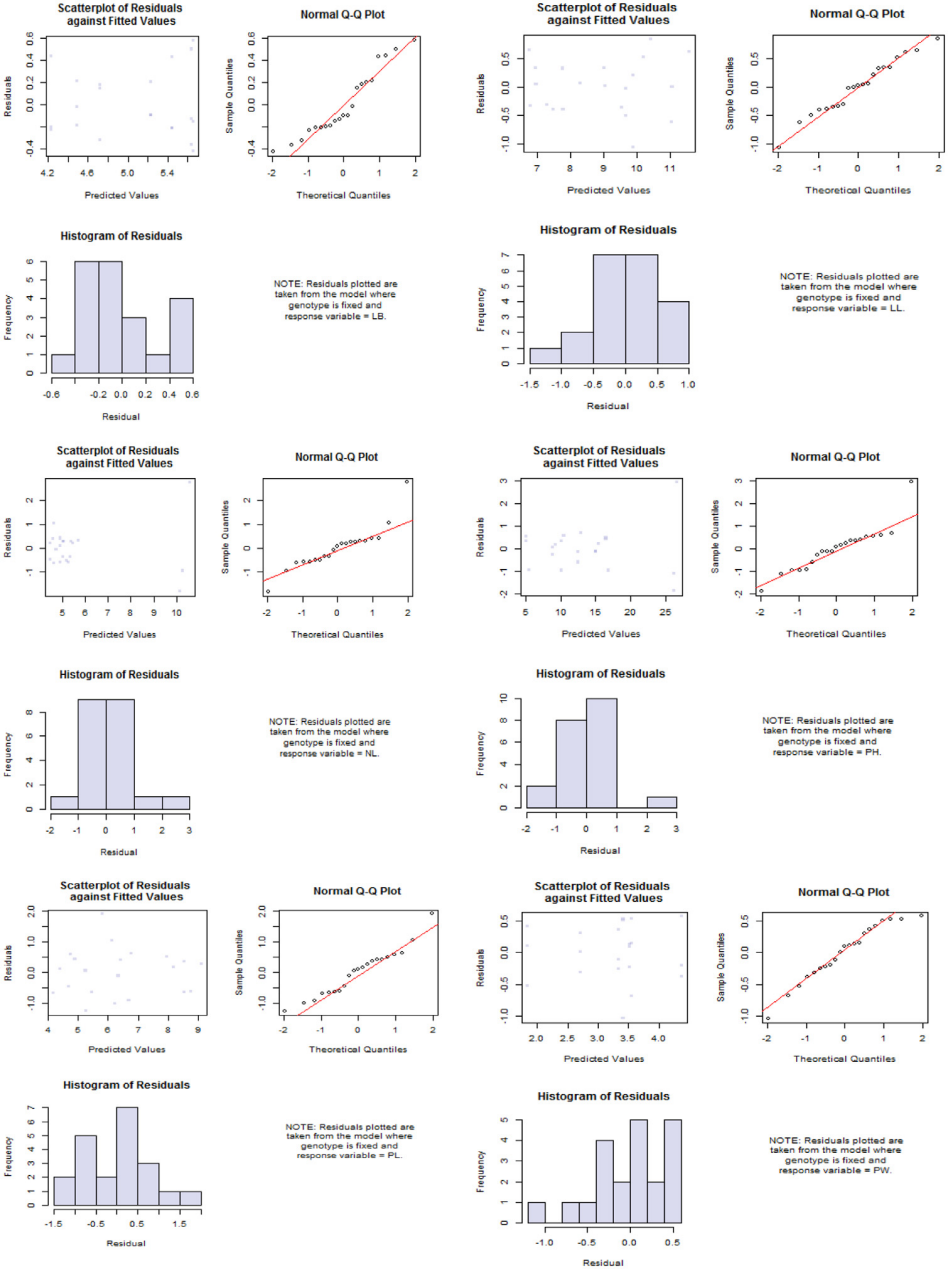

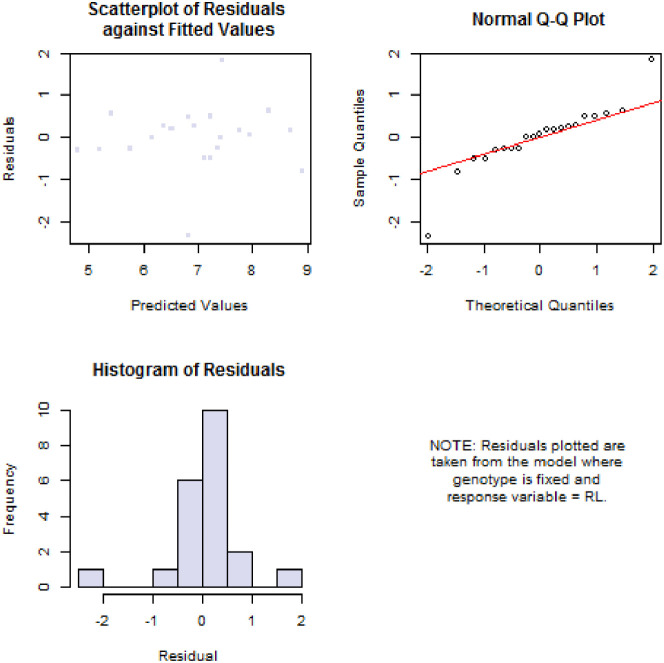
Diagnostic plots of the measured traits. Keys to abbreviations: PH= Plant height (cm), NL= Number of leaves, PL= Petiole length (cm), LL= Leaf length (cm), LB=Leaf breadth (cm), RL=Root length (cm), and PW= Plant weight (g).

Table 1. Contained the results of the analysis of variance (ANOVA) for the investigated data. Box plots showing the measured traits distribution (Fig. 1). Diagnostic plots showing the distribution of the attributes measured (Fig. 2).

**Table 1:** Analysis of variance (ANOVA) for Agronomic traits.

	Mean sum of squares	
Traits	Genotypes (df=6)	Error (df=12)
Plant height (PH)(cm)	138.78***	1.38
Number of leaf(NL)	12.54***	1.17
Petiole length(PL)(cm)	7.88***	0.91
Leaf length(LL)(cm)	7.77***	0.36
Leaf breadth(LB)(cm)	0.99**	0.14
Root length(RL)(cm)	3.75*	0.91
Plant weight(PW)(g)	1.86**	0.29

Keys to abbreviations: df: Degrees of freedom.

Significance. Codes: 0 ‘***’ 0.001 ‘**’ 0.01 ‘*’ 0.05 ‘.’ 0.1 ‘’ 1.

### 1.2 Descriptive Statistic of the Traits

Table 2. presented the descriptive statistics of the data, including the mean, standard deviation, standard error, minimum and maximum values, skewness, kurtosis, and co-efficient of variation.

**Table 2:** Descriptive statistic of the measured traits.

Traits	Mean	Standard Error	Standard deviation	Minimum	Maximum	Kurtosis	Skewness	Co-efficient of variations (CV %)
PH	13.61	2.57	6.8	5.24	26.36	1.56	1.02	8.65
NL	5.75	0.77	2.04	4.67	10.33	6.53	2.53	18.84
PL	6.4	0.61	1.62	4.44	8.8	-1.09	0.47	14.86
LL	8.92	0.61	1.61	6.97	11.23	-1.47	0.04	6.78
LB	5.06	0.22	0.57	4.23	5.66	-1.81	-0.39	7.49
RL	6.96	0.42	1.12	5.13	8.63	0.52	-0.28	13.68
PW	3.25	0.3	0.79	1.84	4.37	1.36	-0.71	16.66

Keys to abbreviations: PH= Plant height (cm), NL= Number of leaves, PL= Petiole length (cm), LL= Leaf length (cm), LB=Leaf breadth (cm), RL=Root length (cm), and PW= Plant weight (g).

### 1.3 Estimation of Genetic Parameters

Table 3. provides a summary of the findings on the genetic parameters of agronomic traits.

**Table 3:** Estimation of genetic parameters agronomic traits.

Traits	σ^2^g	σ^2^p	σ^2^e	GCV	PCV	ECV	hBS	GA	GAM (%)
PH	45.80	47.19	1.39	49.71	50.46	8.65	0.97	13.73	100.89
NL	3.79	4.96	1.17	33.88	38.76	18.84	0.76	3.50	60.99
PL	2.33	3.23	0.91	23.83	28.08	14.86	0.72	2.67	41.64
LL	2.46	2.83	0.36	17.61	18.87	6.77	0.87	3.02	33.87
LB	0.28	0.42	0.14	10.51	12.90	7.47	0.66	0.89	17.66
RL	0.95	1.85	0.91	13.98	19.56	13.67	0.51	1.43	20.60
PW	0.52	0.81	0.29	22.23	27.79	16.67	0.64	1.19	36.64

Keys to abbreviations: σ^2^g= Genotypic variance, σ^2^p= Phenotypic variance, σ^2^e= Environmental variance, GCV=Genotypic co-efficient of variations, PCV=Phenotypic co-efficient of variations, ECV=Environmental co-efficient of variations, hBS= broad‐sense heritability, GA=Genetic advance, GAM=Genetic advance as percent mean, PH= Plant height (cm), NL= Number of leaves, PL= Petiole length (cm), LL= Leaf length (cm), LB=Leaf breadth (cm), RL=Root length (cm), and PW= Plant weight (g).

### 1.4 Association Among Traits

The relationship between the traits that were shown in Tables 4 and 5 is being investigated using the correlation and path analysis.

**Table 4:** Genotypic (G) and phenotypic (P) correlation coefficients among traits.

Traits		PH	NL	PL	LL	LB	RL	PW
PH	G	1	0.91**	0.77*	0.63ns	0.23ns	0.83*	0.04ns
	P		0.76**	0.64**	0.59**	0.20ns	0.52*	0.02ns
NL	G		1	0.80*	0.76*	0.30ns	0.87*	0.18ns
	P			0.67**	0.70**	0.23ns	0.55**	0.18ns
PL	G			1	0.98**	0.83**	0.93**	0.12ns
	P				0.91**	0.60**	0.69**	0.23ns
LL	G				1	0.90**	0.97**	0.23ns
	P					0.69**	0.76**	0.30ns
LB	G					1	0.75ns	0.45ns
	P						0.38ns	0.28ns
RL	G						1	-0.31ns
	P							0.06ns
PW	G							1
	P							

**Keys to abbreviations:** PH= Plant height (cm), NL= Number of leaves, PL= Petiole length (cm), LL= Leaf length (cm), LB=Leaf breadth (cm), RL=Root length (cm), and PW= Plant weight (g). *= significant at 5% of probability, ** = significant at 1% level.

**Table 5:** Path analysis of traits at the genotypic (G) and phenotypic (P) levels demonstrating direct (bold-diagonal) and indirect (off-diagonal) effects (genotypic and phenotypic residual effect was 1.57 and 0.80, respectively).

Traits		PH	NL	PL	LL	LB	RL	Dependent variable (PW) correlation value
PH	G	**-3.00**	0.13	4.65	-0.69	-0.58	-0.47	0.04ns
	P	**-0.27**	0.13	-0.08	0.41	0.00	-0.18	0.02ns
NL	G	-2.72	**0.14**	4.84	-0.83	-0.75	-0.49	0.18ns
	P	-0.21	**0.17**	-0.09	0.49	0.00	-0.19	0.18ns
PL	G	-2.32	0.11	**6.02**	-1.07	-2.09	-0.52	0.12ns
	P	-0.17	0.12	**-0.13**	0.64	0.01	-0.24	0.23ns
LL	G	-1.89	0.11	5.91	**-1.09**	-2.25	-0.55	0.23ns
	P	-0.16	0.12	-0.12	**0.70**	0.01	-0.26	0.30ns
LB	G	-0.69	0.04	5.01	-0.98	**-2.51**	-0.42	0.45ns
	P	-0.05	0.04	-0.08	0.48	**0.01**	-0.13	0.28ns
RL	G	-2.50	0.12	5.58	-1.07	-1.88	**-0.56**	-0.31ns
	P	-0.14	0.10	-0.09	0.53	0.01	**-0.34**	0.06ns

Keys to abbreviations: PH= Plant height (cm), NL= Number of leaves, PL= Petiole length (cm), LL= Leaf length (cm), LB=Leaf breadth (cm), RL=Root length (cm), and PW= Plant weight (g).

### 1.5 Principal Component Analysis

**Fig. 3:**
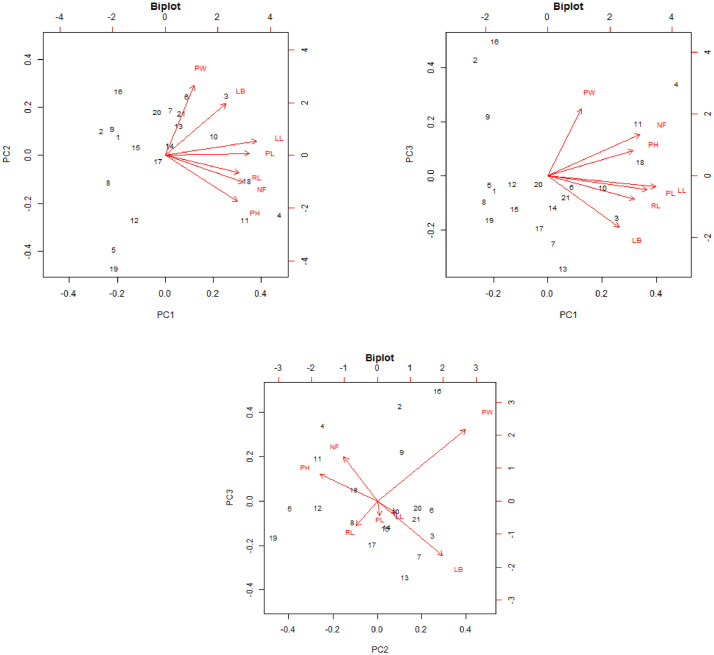
Biplot distribution of principal component based on genotype and traits. Keys to abbreviations: PH= Plant height (cm), NL= Number of leaves, PL= Petiole length (cm), LL= Leaf length (cm), LB=Leaf breadth (cm), RL=Root length (cm), and PW= Plant weight (g).

Fig. 3 depicts a biplot distribution that represents the traits' scores on the principal components of agronomic traits.

### 1.6 Heatmap Analysis

**Fig. 4:**
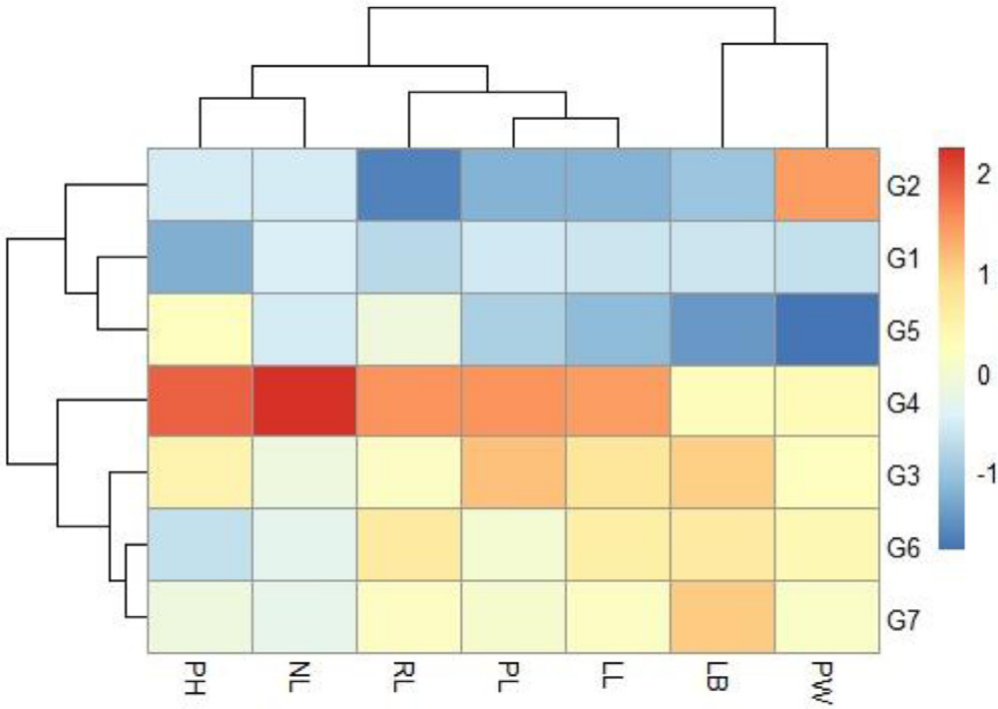
Heatmap displaying the pattern the different genotypes and traits are clustered. Keys to abbreviations: PH= Plant height (cm), NL= Number of leaves, PL= Petiole length (cm), LL= Leaf length (cm), LB=Leaf breadth (cm), RL=Root length (cm), and PW= Plant weight (g).

The heatmap demonstrated how observable traits performed consistently across the germplasm in Fig. 4.

The supplemental file "S1" contains the experiment's raw data. The ANOVA analysis results are shown in supplemental file "S2". The additional file "S3" contains the raw data for Tables 1, 3, 4, 5, and Figs. 1 and 2. In the supplemental file "S4", the raw data for Table 2, Figs. 3, and 4 are displayed. The principal component analysis's analyzed data are shown in the supplemental file "S5". The genotype image is shown in the supplemental file "S6."

## 2 Experimental Design, Materials and Methods

During Kharif-1 season, the experiment was conducted at the Regional Station of the Bangladesh Institute of Research and Training on Applied Nutrition (BIRTAN), in Rangpur, Bangladesh. The tista meander floodplain, which covers an area of 946,803 ha, primarily consists of high and medium high land areas. The soils in this area have moderately acidic soils (pH 4.6–6.5), low organic matter contents (1%) on higher ground, and moderate organic matter contents (2%) on lower ground. Overall, fertility levels are low to medium, while K and CEC status are often in the middle of the scale. In general, soils are good in holding water [2,3].

In this research, popular leafy vegetables called Napa shak (*Malva parviflora*) from the Rangpur region of Bangladesh were used. A total of 7 genotypes were used in this experiment. Randomized complete block design (RCBD) with three replication were used as experimental design. Direct seeding was carried out in pot. Date of seeding was April 1, 2021. The plastic pot had a 10 inch diameter and 10 inch height, and could hold 10 kg of soil. The leafy vegetable cheeseweed (*Malva parviflora* L.) is an orphan leafy vegetables. Chemical fertilizers weren't used. To prepare the soil, just vermicompost (25 percent vermicopost and 75 percent soil) was applied. Irrigation, weeding, and thinning were practiced as per requirements.

The R statistics platform (version 4.1.2 of the program) was used to conduct the statistical studies [4]. Using the R package "variability", analyses of variance (ANOVA), estimation of genetic parameters, and traits association analysis were performed. The formula presented by [5] was used to compute the phenotypic and genetic variance, as well as the genotypic and phenotypic coefficients of variation (GCV and PCV). Heritability in the broad sense (hBS) and genetic advance (GA) calculation as described in [6], [7], [8], [9], [10], [11]. "Pheatmap" was used to create a heatmap diagram. The Statistical Tool for Agricultural Research (STAR) software, version 2.0.1(2014), carried out principal component analysis. Utilizing plant breeding tools (PBTools) Version: 1.3, box plots and diagnostic plots were created.

## Ethics Statements

Not applicable

## Funding

This research did not receive any specific grant from funding agencies in the public, commercial, or not-for-profit sectors.

## CRediT authorship contribution statement

**Md. Marufur Rahman:** Conceptualization, Methodology, Visualization, Data curation, Writing – original draft. **Md. Ashraful Alam:** Validation, Investigation, Writing – review & editing. **Hasibul Hasan:** Data curation, Software. **Mirana Akhter Sumi:** Writing – review & editing. **Md. Maksudul Haque:** Visualization, Investigation, Writing – review & editing.

## Declaration of Competing Interest

The authors declare that they have no known competing financial interests or personal relationships that could have appeared to influence the work reported in this paper.

## Data Availability

Dataset on genetic variation and trait association in cheeseweed (Malva parviflora L.) genotypes for agronomic traits (Original data) (Mendeley Data). Dataset on genetic variation and trait association in Malva parviflora genotypes for agronomic traits (Original data) (Mendeley Data).

## Appendix Supplementary materials

Image, application 1
