# Supplementary material for: Dataset on genetic variation and trait association in cheeseweed (Malva parviflora L.) genotypes for agronomic traits
Source: Data Brief. 2022 Oct 11;45:108651. doi: 10.1016/j.dib.2022.108651 (PMC9679472; doi:10.1016/j.dib.2022.108651)
Supplement: Supplementary file 1 [file mmc1.zip › S2.docx]

**Analysis of variance (ANOVA)**

**Plant height (PH)**

Df Sum Sq Mean Sq F value Pr(>F)

Replication 2 4.67 2.333 1.6822 0.227

Genotype 6 832.72 138.787 100.0600 1.533e-09 ***

Residuals 12 16.64 1.387

**Number of leaf (NL)**

Df Sum Sq Mean Sq F value Pr(>F)

Replication 2 4.011 2.0053 1.7111 0.2219334

Genotype 6 75.238 12.5397 10.6998 0.0003198 ***

Residuals 12 14.063 1.1720

**Petiole length (PL)**

Df Sum Sq Mean Sq F value Pr(>F)

Replication 2 3.886 1.9432 2.1467 0.1595905

Genotype 6 47.303 7.8839 8.7095 0.0008386 ***

Residuals 12 10.862 0.9052

**Leaf length (LL)**

Df Sum Sq Mean Sq F value Pr(>F)

Replication 2 2.512 1.2562 3.4364 0.06608

Genotype 6 46.651 7.7751 21.2688 9.689e-06 ***

Residuals 12 4.387 0.3656

**Leaf breadth (LB)**

Df Sum Sq Mean Sq F value Pr(>F)

Replication 2 0.0712 0.03561 0.2493 0.783280

Genotype 6 5.9546 0.99243 6.9483 0.0002284 ***

Residuals 12 1.7140 0.14283

**Root length (RL)**

Df Sum Sq Mean Sq F value Pr(>F)

Replication 2 4.1667 2.0833 2.2992 0.14279

Genotype 6 22.4984 3.7497 4.1383 0.01746 *

Residuals 12 10.8733 0.9061

**Plant weight (PW)**

Df Sum Sq Mean Sq F value Pr(>F)

Replication 2 0.2838 0.14191 0.4838 0.627958

Genotype 6 11.1454 1.85757 6.3328 0.003382 **

Residuals 12 3.5199 0.29333

Signif. codes: 0 ‘***’ 0.001 ‘**’ 0.01 ‘*’ 0.05 ‘.’ 0.1 ‘ ’ 1

Keys to abbreviations: PH= Plant height (cm), NL= Number of leaves, PL= Petiole length (cm), LL= Leaf length (cm), LB=Leaf breadth (cm), RL=Root length (cm), and PW= Plant weight (g)
