# Supplementary material for: Dataset on genetic variation and trait association in cheeseweed (Malva parviflora L.) genotypes for agronomic traits
Source: Data Brief. 2022 Oct 11;45:108651. doi: 10.1016/j.dib.2022.108651 (PMC9679472; doi:10.1016/j.dib.2022.108651)
Supplement: Supplementary file 1 [file mmc1.zip › S5.docx]

**Principal component analysis analyzed data**

| Statistics | PC1 | PC2 | PC3 | PC4 | PC5 | PC6 | PC7 |
| --- | --- | --- | --- | --- | --- | --- | --- |
| Standard deviation | 1.98 | 1.09 | 0.89 | 0.70 | 0.54 | 0.49 | 0.27 |
| Proportion  of  Variance | 0.56 | 0.17 | 0.11 | 0.07 | 0.04 | 0.03 | 0.01 |
| Cumulative  Proportion | 0.56 | 0.73 | 0.84 | 0.91 | 0.96 | 0.99 | 1.00 |
| Eigen Values | 3.92 | 1.18 | 0.80 | 0.49 | 0.29 | 0.24 | 0.07 |

| EIGENVECTORS | | | | | | | |
| --- | --- | --- | --- | --- | --- | --- | --- |
| Variables | PC1 | PC2 | PC3 | PC4 | PC5 | PC6 | PC7 |
| PH | 0.38 | -0.44 | 0.25 | -0.33 | 0.05 | -0.68 | -0.16 |
| NL | 0.41 | -0.26 | 0.41 | -0.04 | -0.53 | 0.44 | 0.35 |
| PL | 0.44 | 0.02 | -0.13 | -0.32 | 0.70 | 0.32 | 0.29 |
| LL | 0.48 | 0.14 | -0.11 | 0.07 | -0.09 | 0.28 | -0.80 |
| LB | 0.32 | 0.50 | -0.50 | -0.28 | -0.42 | -0.28 | 0.27 |
| RL | 0.39 | -0.16 | -0.23 | 0.82 | 0.11 | -0.21 | 0.21 |
| PW | 0.15 | 0.67 | 0.66 | 0.17 | 0.16 | -0.19 | 0.05 |

**Keys to abbreviations:**  PH= Plant height (cm), NL= Number of leaves, PL= Petiole length (cm), LL= Leaf length (cm), LB=Leaf breadth (cm), RL=Root length (cm), and PW= Plant weight (g)


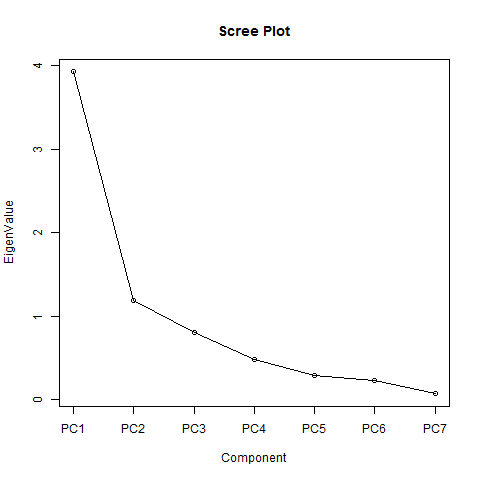


Fig. Scree plot of principal component analysis
