# Supplementary material for: Dataset on genetic variation and trait association in cheeseweed (Malva parviflora L.) genotypes for agronomic traits
Source: Data Brief. 2022 Oct 11;45:108651. doi: 10.1016/j.dib.2022.108651 (PMC9679472; doi:10.1016/j.dib.2022.108651)
Supplement: Supplementary file 1 [file mmc1.zip › S6.docx]

**Image of genotypes**

| **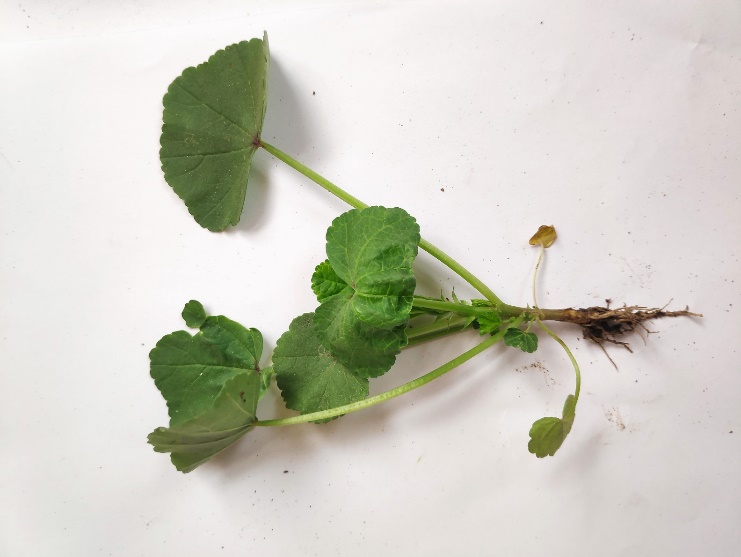** | **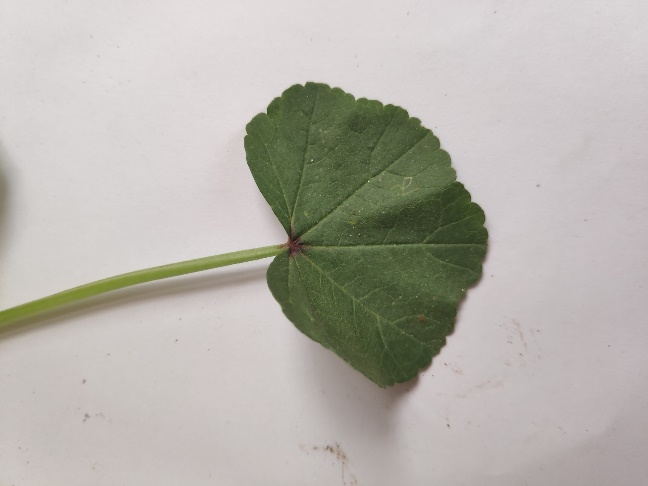** |
| --- | --- |
| **Genotype 1(G1)** | |
| **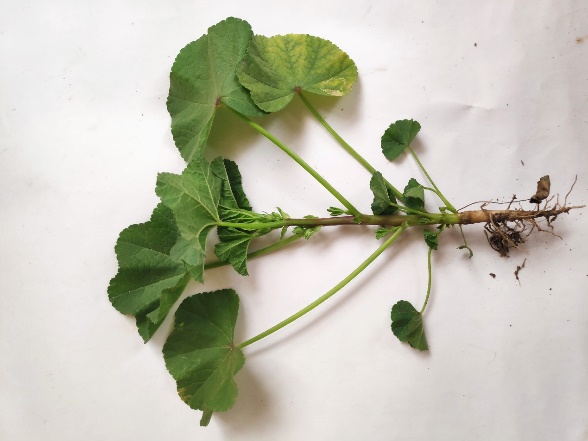** | **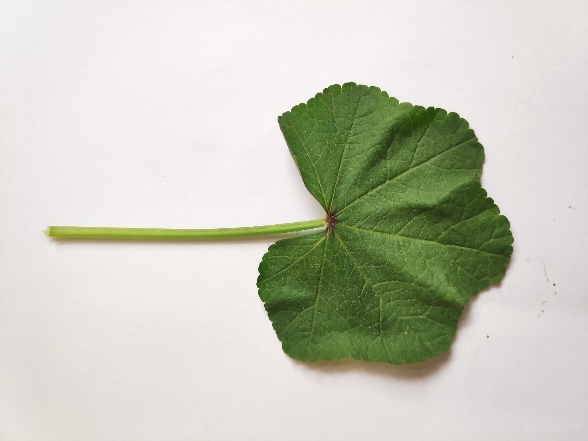** |
| **Genotype 2 (G2)** | |
| **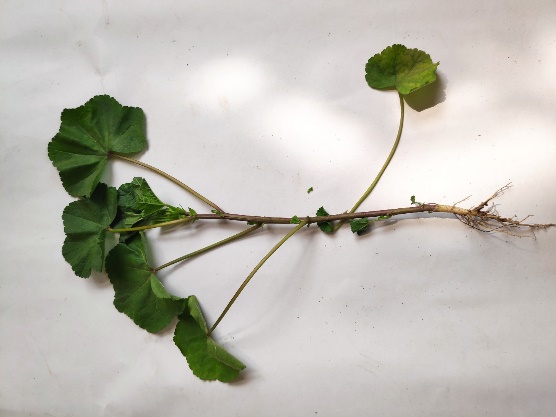** | **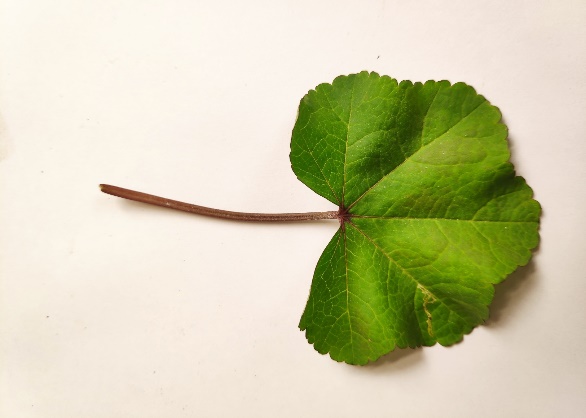** |
| **Genotype 3 (G3)** | |
| **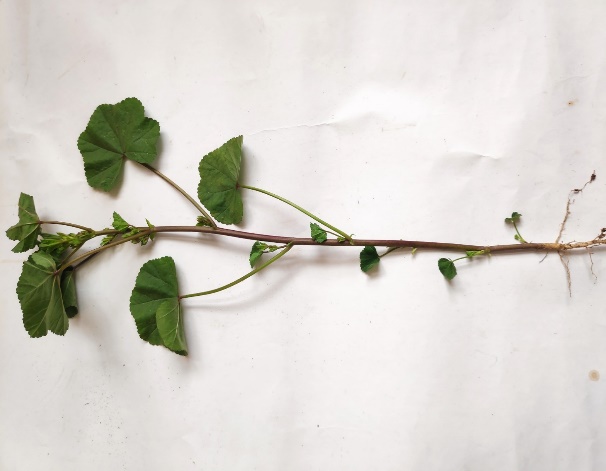** | **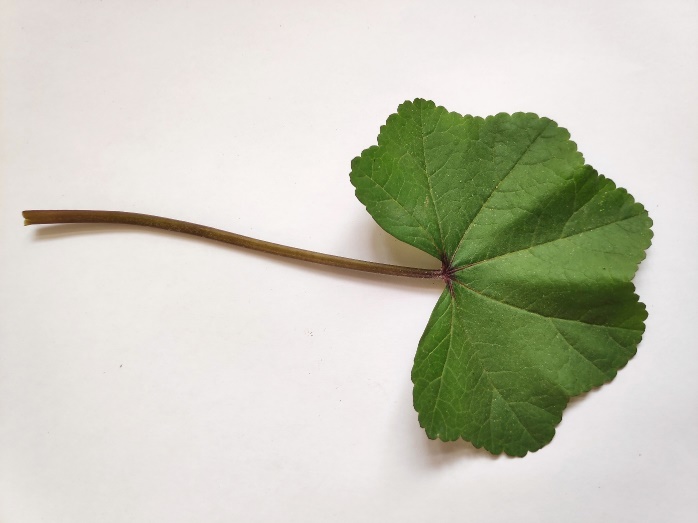** |
| **Genotype 4 (G4)** | |
| **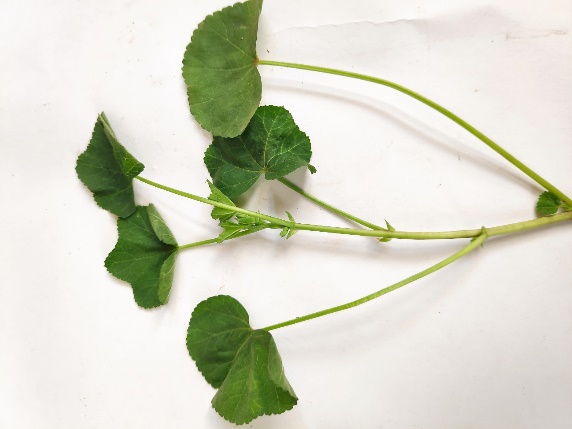** | **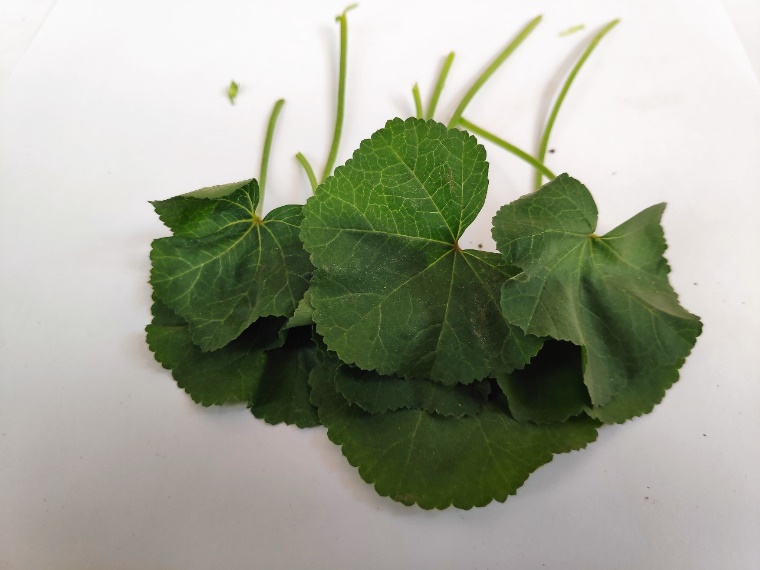** |
| **Genotype 5 (G5)** | |
| **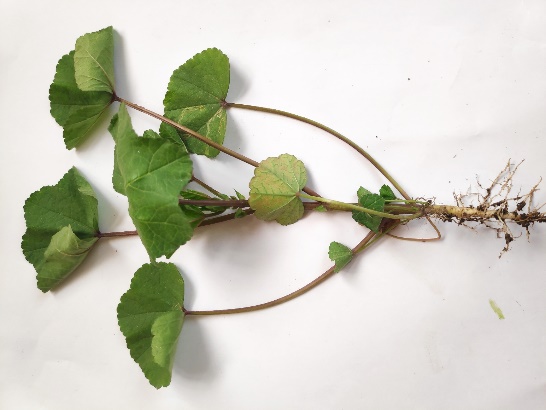** | **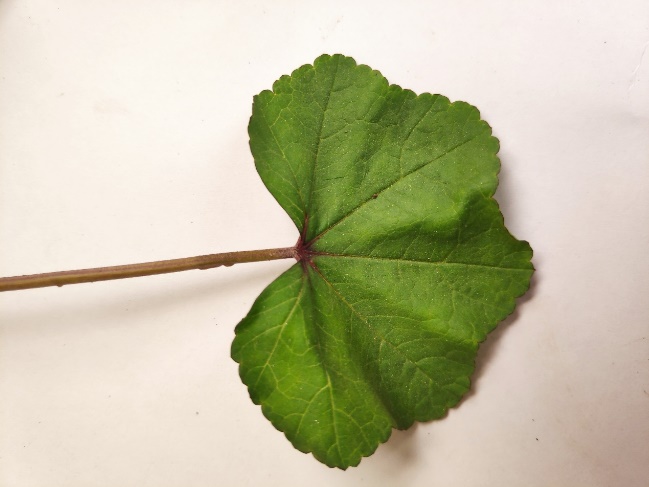** |
| **Genotype 6 (G6)** | |
| **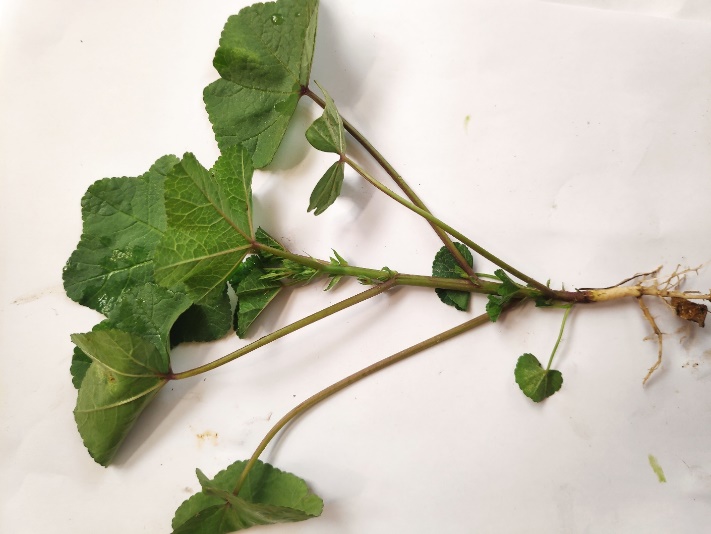** | **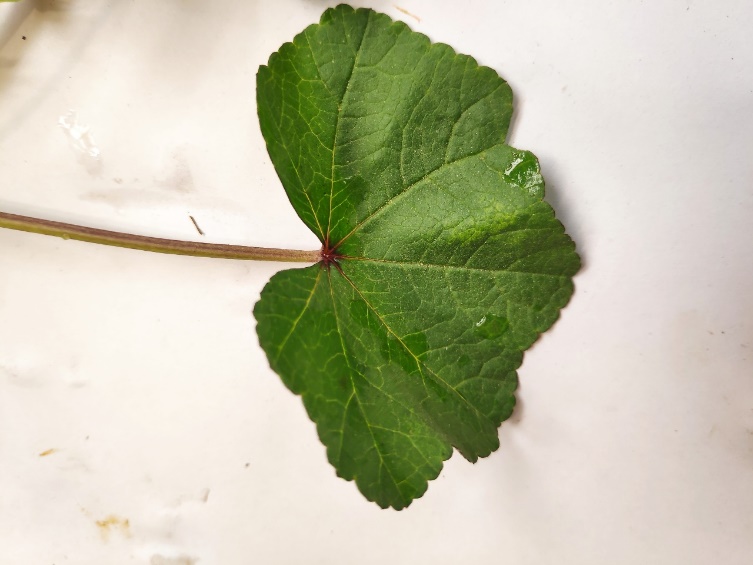** |
| **Genotype 7 (G7)** | |

**Image of genotypes were taken by Poco Phone F1 Mobile device.**
